# Familial Aggregation between the 14^th^ and 21^st^ Century and Type 2 Diabetes Risk in an Isolated Dutch Population

**DOI:** 10.1371/journal.pone.0132549

**Published:** 2015-07-20

**Authors:** Kees L. de Visser, Gijs W. D. Landman, Betty Meyboom-de Jong, Wim de Visser, Gerard J. te Meerman, Henk J. G. Bilo

**Affiliations:** 1 Diabetes Centre, Isala, 8025 BT, Zwolle, The Netherlands; 2 Department of General practice, University Medical Center Groningen and University of Groningen, 9700 RB, Groningen, The Netherlands; 3 General practice Urk, 8322 BA, The Netherlands; 4 Department of internal medicine, Gelre hospital, 7334 DZ, Apeldoorn, The Netherlands; 5 Langerhans Medical Research Institute, Zwolle, The Netherlands; 6 Department of Internal Medicine, University Medical Center Groningen and University of Groningen, 9700 RB, Groningen, The Netherlands; 7 Department of genetics, University Medical Center Groningen and University of Groningen, 9700 RB, Groningen, The Netherlands; University of Birmingham, UNITED KINGDOM

## Abstract

**Introduction:**

The development of type 2 diabetes results from an interaction of hereditary factors and environmental factors. This study aimed to investigate the contribution of interrelatedness to the risk of developing type 2 diabetes in an isolated Dutch population.

**Materials and Methods:**

A genealogical database from inhabitants living on the former island Urk between the 14^th^ and 21^st^ century was constructed. In a case-control study, effects of interrelatedness and the risk of type 2 diabetes were estimated with Kinship Coefficients (KCs). Relative risks in first, second, and third degree relatives and spouses of inhabitants with type 2 diabetes were compared to matched controls.

**Results:**

Patients with type 2 diabetes were more interrelated, expressed by a higher KC compared to controls (7.2 vs. 5.2, p=0.001). First, second and third degree relatives had an increased risk of developing type 2 diabetes. Second degree relatives had a similar risk,1.7 (1.5-2.0) as third degree relatives,1.8 (1.5-2.2). Spouses of patients with diabetes had a 3.4 (2.7-4.4) higher risk of developing type 2 diabetes.

**Conclusions:**

Interrelatedness was higher among inhabitants with type 2 diabetes compared to controls. This differences extended beyond the nuclear family, thereby supporting the hypothesis that interrelatedness contributed to the development of type 2 diabetes on Urk. However, the size of this effect was small and the patterns of risk in first, second and third degree relatives suggested that factors other than interrelatedness were the main contributors to the development of type 2 diabetes on Urk.

## Introduction

Both environmental factors and hereditary factors contribute to the development of type 2 diabetes. On a population level, environmental factors can to a large extent explain the dramatic rise in prevalence of type 2 diabetes. During the Second World War, type 2 diabetes was virtually non-existent in the Netherlands [1] and the increase in prevalence of type 2 diabetes thereafter shows a direct parallel with the increased prevalence of obesity and a sedentary lifestyle, both major risk factors for developing type 2 diabetes [2]. Still, a substantial proportion of people with obesity do not develop type 2 diabetes, suggesting that there is an interaction between environmental and hereditary factors [3]. No single gene or gene-lifestyle interaction of overwhelming importance has been linked to the development of type 2 diabetes [4–8].

An increased familial aggregation of type 2 diabetes has been reported in various aggregation studies. Familial aggregation is a reflection of a variety of processes; ranging from genetic influences, family environment, intra-uterine and epigenetic processes [9]. In parental studies, offspring and siblings from parents with type 2 diabetes had an increased risk of type 2 diabetes [8–13]. High concordance rates were reported between twins and in twins reared apart childhood environment did not have a large effect on body mass index later in life [14–18]. Parental and twin studies can be subject to incomplete case ascertainment [8,10,18].

An alternative method of studying the contribution of heredity factors to the development of a disease is a pedigree study. This design has been used before to study the contribution of heredity to the development of rheumatoid arthritis on Iceland [19]. In pedigree studies, family members outside the nuclear family are included. Beyond the nuclear family, the effect of shared family environment decreases.

This pedigree study was performed in a formerly isolated fisherman’s population living on the island Urk between the 14th and 21st century [20,21]. With a case-control design, using genealogical data of a whole population, we aimed to study to what extent interrelatedness contributed to the development of type 2 diabetes on Urk.

## Materials and Methods

### The population on Urk

For many centuries, the population on Urk have lived in isolation. Before 2001, immigration was limited and often temporarily; it was not until 1938 that the island of Urk was connected to the mainland by a dike. The inhabitants of Urk share a strong identity, both in religious and social aspects. The majority of the approximately 48,930 inhabitants who live and have lived on Urk up to 2001 descended from a small group of founders. A plague epidemic in 1637 killed the majority of the population; only 151 survived.

In 1990, the general practitioners on Urk observed an increased age-adjusted prevalence of type 2 diabetes, at that time 3.1% compared to 1.8% in the overall Dutch population [20,21]. Thereafter, a genealogy database was constructed going back to the beginning of the 14th century.

### The genealogy database

The genealogy information was traced with the use of vital records, parish records, municipality records and other available documentation. All inhabitants were given an identification number, date of birth and death, and identifiers of father and mother. This resulted in a combined cohort of 48,930 inhabitants who were born, married or died in the municipality of Urk. The database included all inhabitants of Urk up to 2001 going back to the year 1300. From the 151 plague survivors in 1637, 105 (69.5%) were definitely identified through death certificates. The remaining 46 were not identified as survivors and were (most likely) included in the database through other records, for example through marital records.

The founders of all individual ancestries of all inhabitants, including those with T2DM and the controls, were identified. A founder was defined as a person in the pedigree from whom no parent could be specified.

The study was conducted in accordance with the regulations and laws regarding studies and privacy that applied before 2001 and in accordance to the principles expressed in the in the Declaration of Helsinki. The data needed for this study were extracted by the present owner of the database (Brouwer, Urk), and after de-identification provided to the researchers. A special commission appointed by the local community gave permission for this study. The presented research on data from Urk was carried out before it became mandatory, as from 2011 in the Netherlands, to submit all research proposals including non-WMO related research (WMO = “Wet medisch-wetenschappelijk onderzoek met mensen”: Law on medical scientific research in humans) for assessment to a medical ethical committee (METC). Before this decision, there was no obligation in the Netherlands to formally submit non-WMO related research to a METC. Therefore, according to Dutch rules and laws, no formal METC rules were infringed upon when using the anonymized Urk data. The METC of the Isala Hospital, the Netherlands, has retrospectively approved that this study was not WMO related, see S1 File.

### Cases and controls

Cases were defined as patients with type 2 diabetes known to the general practitioners who lived on Urk in the period from 1996 to 2000. The diagnosis of type 2 diabetes was based on criteria as described in the national guidelines of the Dutch College of General Practitioners from that time period [20,21]. All patients in the Netherlands have a primary care physician.

Case were matched (1:1) according to the absence of type 2 diabetes, concordance of gender, birth year, birth month and number of parents and grandparents in the database. Matching for the number of parents and grandparents was done to increase comparability regarding missing and available genealogical data and degree in which ancestries originated from Urk.

### Kinship coefficients

**Fig 1:**
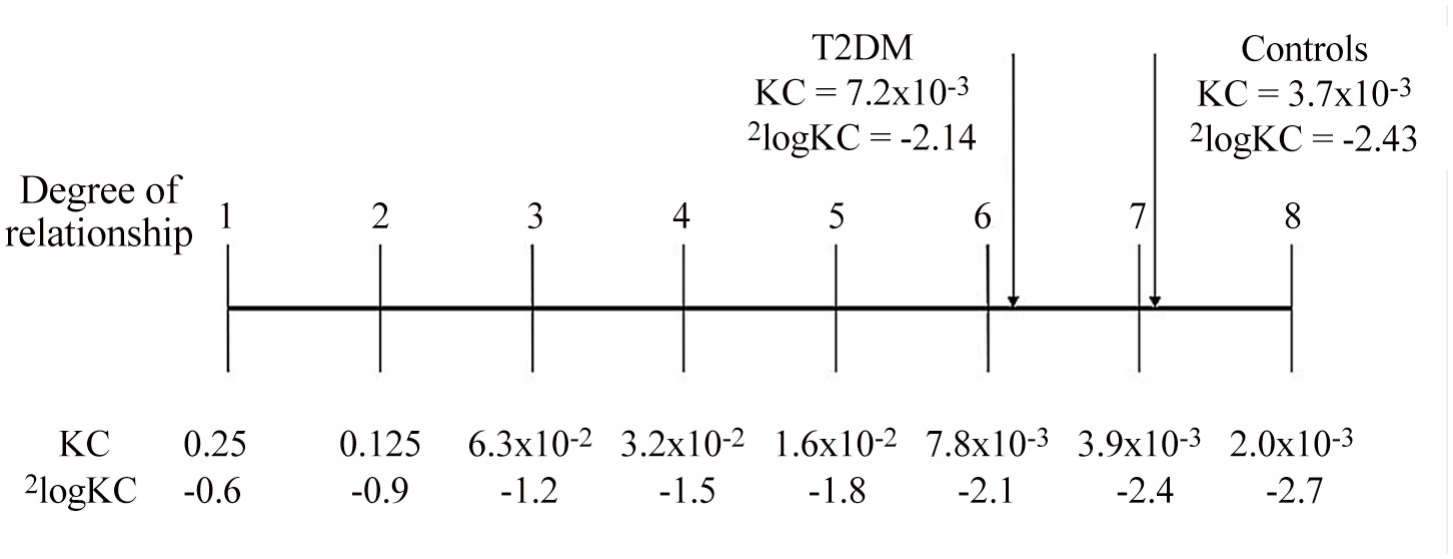
Mean kinship coefficients (KC) of inhabitants with type 2 diabetes and controls on a scale representing differences in degree of relationship (^2^logKC).

For the analysis of heredity of type 2 diabetes, kinship coefficients (KCs) were calculated. The KC is defined as the probability that a random allele from two persons is inherited from a common ancestor [22]. The probability that one parent will pass an identical gene to two offspring is ½; each individual carries two sets of genes, one paternal and one maternal. Assuming non-consanguinity, the KC is 1/4 for first-degree relatives, 1/8 for second-degree relatives and so on, each value being half the expected fraction of the genome shared by these relatives. Differences in KCs can be interpreted using Fig 1.

### Risk ratio’s in relatives and spouses

The risk-ratio’s for relatives of persons with type 2 diabetes were derived from the number of relatives with type 2 diabetes divided by the total number of relatives, divided by the prevalence in the population. The calculation of risk-ratio’s of type 2 diabetes in relatives, were restricted to relatives born in the period between 1900 and 1960, since the majority of persons in the Netherlands develop type 2 diabetes at an older age. A difference in risk for siblings compared to genetically equally related first-degree relatives points to effects other than hereditary factors, for example effects of shared environment, that explain the increased risk of developing type 2 diabetes. The same holds true for an increased risk ratio in spouses.

### Sensitivity analyses

Three sensitivity analyses were carried out. The first aimed to control for shared environment in the nuclear family; by calculating KCs after excluding first- and second-degree related pairs. The second aimed to give insight in the degree of interrelatedness, by excluding all unrelated pairs. The third investigated whether a possible increased risk for developing type 2 diabetes in spouses was actually caused by increased inbreeding; by calculating the mean KC between spouses.

### Statistical analysis

The KC is the sum of probabilities that two persons share the same identical allele, using all possible disjoint pathways in the genealogy database. The KC is calculated with a path-searching algorithm where all possible connections between pairs are weighted inversely proportional to 0.5 to the power k, where k is the length of the path. There are several pathways within the genealogy database that theoretically connect two persons, the mean KC was calculated by averaging the KC of all possible pair-wise combinations of inhabitants. To test for differences in relatedness among the inhabitants with and without type 2 diabetes. The mean KCs were calculated for all inhabitants with type 2 diabetes. These were compared with the mean KC for the matched control group. For testing relatedness in cases compared to controls a *t*-test with estimation of separate variances through *U* statistics was used. An exact calculation method was developed to calculate the variance of the mean of all pairs, using the theory of *U* statistics. Testing the risk-ratio was done using the 95% confidence interval for likelihood ratios

## Results

The total number of persons with type 2 diabetes in the period 1996 to 2000 known to their primary care physicians was 602. In the group with type 2 diabetes, 49 inhabitants could not be connected to another person through a common ancestor, compared to 78 inhabitants in the matched control group.

From the persons born in the period between 1900 and 1960, 7.9% (825 / 10,433) descended from parents outside Urk. A total of 24% of inhabitants with type 2 diabetes were completely unrelated. A total number of 1145 founders were found for the combined groups of inhabitants with type 2 diabetes and controls. From the 1145 founders, 150 were founder for the group with type 2 diabetes only, 432 were founders for the control group only, and 563 were founder for both groups. The mean year of birth for founders was 1678 AD (SD 57, range 1300–1764). The common founders were found on average more often in the ancestries of inhabitants with type 2 diabetes than in controls. None of the founders was identified as being an excessively more prominent founder in the group with type 2 diabetes. The difference in percentages in which the common founders were ancestors of both groups was significant (*t*-test: *t* = 29·5; p<0·000). The slope of the fit-line (β = 1·22; 95%CI: 1·19–1·24). Meaning that common founders were present more frequently in ancestries of inhabitants with type 2 diabetes than in controls.

### Kinship coefficients

The median interrelatedness was a seventh-degree relationship (range 5–13). The interrelatedness among inhabitants with type 2 diabetes was higher compared to the matched control group, mean KC (7.1x10^-3^) and (3.7x10^-3^), respectively, Table 1.

**Table 1:** Kinship coefficients of inhabitants with type 2 diabetes (n = 519) and controls (n = 519).

	KC for type 2 diabetes *	KC for controls *	P-value
All inhabitants	7.2 SD 0.1	3.7 SD 0.1† / 5.2 SD 0.1‡	<0.001† / <0.001‡
Excluding first-degree related pairs	6.6 SD 0.1	4.8 SD 0.1	<0.001
Excluding first- and second-degree related pairs	6.4 SD 0.1	4.6 SD 0.1	<0.001
Excluding unrelated inhabitants	9.2 SD 0.2	7.6 SD 0.2	<0.001

* The KC and SD values are multiplied by 1,000.

^†^ controls randomly selected

^‡^ controls matched for same numbers of parents and grandparents in database

The results from the three sensitivity analyses were consistent. The KC after excluding all first-degree related pairs (4.8x10^-3^), all first- and second-degree related pairs (4.6x10^-3^) and all unrelated inhabitants (7.6x10^-3^), was lower in the control group compared to inhabitants with type 2 diabetes. After excluding unrelated inhabitants, the significant difference in mean KC remained present in inhabitants with type 2 diabetes compared to controls. Matching for number of parents and grandparents in the database did not result in relevantly different results.

The mean KC between the 417 spouses of inhabitants with type 2 diabetes was 4.1x10^-3^ (SD 0.2x10^-3^), which was comparable with the mean KC of 3.9x10^-3^ (SD 0.2x10^-3^) of the spouses of controls.

### Risk Ratio’s for developing type 2 diabetes in relatives and spouses

The risk-ratios for first-degree related siblings and offspring, for second-degree related uncles/aunts and nephew/nieces as well as for third-degree related cousins were significantly increased, Table 2. The relative risk for siblings was 5.1 (4.6–5.8), which was higher than that of genetically equally related offspring, 2.8 (2.2–3.5). Spouses had a significantly increased risk-ratio for developing type 2 diabetes, 3.4 (2.7–4.4).

**Table 2:** Estimated risk ratios for the relatives of inhabitants with type 2 diabetes.

Relative*	No. of affected relatives	Total no. of relatives	Prevalence in relatives (%)	Risk ratio (95% CI)^#^
Offspring	51	395	12.9	2.8 (2.2–3.5)
Siblings	480	2007	23.9	5.1 (4.6–5.8)
Uncles / aunts	131	1348	9.7	2.1 (1.8–2.5)
Nephews / nieces	130	1538	8.4	1.8 (1.5–2.2)
Cousins	457	5755	7.9	1.7 (1.5–2.0)
Spouses	66	417	15.8	3.4 (2.7–4.4)
Husbands	36	214	16.4	3.5 (2.5–4.9)
Wives	30	203	4.8	3.3 (2.3–4.8)

* Year of birth: 1900–1960.

^#^The denominator for calculating the relative risk is the prevalence in the total population born in the same time period. The prevalence for the total population is 0.046 (480/10,433), for men 0.048 (243/5080) and for women 0.044 (237/5353).

## Discussion

In this historically isolated Caucasian population, inhabitants with type 2 diabetes were more interrelated than matched controls. The risk of developing type 2 diabetes was increased in first degree relatives. The increased risk of type 2 diabetes extended beyond the nuclear family. The extent to which interrelatedness contributed to the risk of developing type 2 diabetes on Urk was small. The similar risk in second and third degree relatives and the high risk in spouses suggest that factors other than interrelatedness explain the increase in prevalence of type 2 diabetes on Urk.

To our knowledge, this is the first study in a historically isolated population with many centuries of genealogic data that investigated the relationship between familial aggregation and type 2 diabetes. Previous familial aggregation studies on type 2 diabetes mostly focused on first-degree relatives and were subject to a risk for incomplete case ascertainment or over-reporting [10,14,16,23,24]. In the present study, information on relatedness from the whole population was available to estimate the risk of type 2 diabetes in relatives beyond the nuclear family.

The risk-ratio in siblings for developing type 2 diabetes was substantially higher compared to offspring, despite their equal probability of sharing a random allele. The difference in risk of developing type 2 diabetes between equally related offspring’s and siblings could be explained by factors other than hereditary factors [4,25]. For example, (un)healthy eating patterns in childhood are probably shared more intensively between siblings compared to offspring. Next to a difference in lifestyle, other factors like starvation periods or climate changes, could have provoked hereditary epigenetic changes that possibly led to an alteration in the risk of diabetes. Interrelatedness is a reflection of more than just genetic processes; together with family environment, intra-uterine and epigenetic processes, patterns of enhanced intra-familial phenotypic resemblance are generated [9].

There was an interesting similarity in risk of type 2 diabetes in family members beyond the nuclear family of inhabitants with type 2 diabetes. The risk ratios for type 2 diabetes in cousins compared to nephews and nieces were more or less comparable, despite that they have a different probability of sharing a random allele. A possible explanation for this difference could be a difference in exposure to (family) environmental factors. This hypothesis is consistent with the recently proposed intergenerational prevention framework for type 2 diabetes, with emphasis on exposure to environmental factors in early life as risk factors for developing type 2 diabetes and as a basis for new type 2 diabetes prevention strategies [12].

The remarkably high risk-ratio in spouses suggested that environmental factors in later life also influenced the risk of type 2 diabetes. An increased risk of developing diabetes in spouses was reported before [26]. Possible explanations for this spousal concordance are increased awareness of symptoms by spouses and or an increased likelihood of being tested. Potential other explanations are; the distinct social structure on Urk, shared (un)healthy behaviour and ‘assortative mating’; obese people theoretically could be more likely to marry an obese partner. Unfortunately, data on BMI (at the date of marriage), food intake and exercise were not available in this study. There was no evidence for the presence of inbreeding and therefore inbreeding would be an unlikely explanatory factor for the spousal concordance.

Strengths of this study were the inclusion of a total population and the matched case-control design. The main limitation was the incompleteness of the genealogy database; not all ancestry lines extended to the survivors of the plague epidemic. Furthermore, it was impossible to validate all connections between persons, and to obtain information on illegitimate births or false paternity. Incomplete knowledge of ancestry lines could have resulted in an underestimation of the degree of relatedness. Fortunately, the distribution of missing data was not different among inhabitants with and without type 2 diabetes making relevant differences in missing genealogical data unlikely. The mean KCs in inhabitants with type 2 diabetes compared to controls, matched for age, gender and the number of (grand) parents in the database supported the validity of the main results. Furthermore, the construction and verification of the database took a considerable amount of time and no clinical data were available after 2001.

In conclusion, based on 7 centuries of familial aggregation in an isolated population, interrelatedness contributed to the development of type 2 diabetes. Although other factors than interrelatedness probably were the main contributors to the risk of type 2 diabetes on Urk. These results could potentially be of importance when developing type 2 diabetes prevention strategies.

## Supporting Information

S1 File Retrospective approval by the Medical Ethics Committee of the Isala Hospital that this study was not WMO related. (PDF)
