# Correction: Familial Aggregation between the 14^th^ and 21^st^ Century and Type 2 Diabetes Risk in an Isolated Dutch Population

**DOI:** 10.1371/journal.pone.0137939

**Published:** 2015-09-04

**Authors:** The PLOS ONE Staff

Dr. Nanne Kleefstra should be listed as the third author, and Dr. Kleefstra’s affiliations are 1: Diabetes Centre, Isala, 8025 BT, Zwolle, The Netherlands, 5: Langerhans Medical Research Institute, Zwolle, The Netherlands, and 6: Department of Internal Medicine, University Medical Center Groningen and University of Groningen, 9700 RB, Groningen, The Netherlands. The contributions of this author are as follows: conceived and designed the experiments.

The correct citation is: de Visser KL, Landman GWD, Kleefstra N, Meyboom-de Jong B, de Visser W, te Meerman GJ, et al. (2015) Familial Aggregation between the 14th and 21st Century and Type 2 Diabetes Risk in an Isolated Dutch Population. PLoS ONE 10(7): e0132549. doi:10.1371/journal.pone.0132549

The publisher apologizes for this error.
