# Supplementary material for: Familial Aggregation between the 14th and 21st Century and Type 2 Diabetes Risk in an Isolated Dutch Population
Source: PLoS One. 2015 Jul 20;10(7):e0132549. doi: 10.1371/journal.pone.0132549 (PMC4508118; doi:10.1371/journal.pone.0132549)
Supplement: S1 File — (PDF) [file pone.0132549.s001.pdf]

Postadres:
METC Isala
Gebouw Mondriaan, kamer 0.25
Postbus 10400
8000 GK Zwolle
Bezoekadres:
Dr. van Deenweg 1-11, Zwolle
Gebouw Mondriaan, kamer P0.25
Tel 038-4243082
E-mail METC@isala.nl
Info www.isala.nl

De weledelzeergeleerde
Professor dr. Bilo
Diabetes centrum
Isala

Zwolle 10-02-15,

Changes in METC Isala rules as of September 2011

Dear professor Bilo,

Please find attached the letter of the board of directors of the Isala hospital.

In this letter it is announced that all research including the non-WMO related research has to be submitted for assessment to the medical ethical committee (METC/IRB) as of 6th September 2011.

Before this date, there was no obligation to formally submit non-WMO related research to an accredited METC. A non-WMO study carried out before 2011 was, and still is, not considered a breach of any rule or law.

The Isala has special considerations with these non-WMO studies carried out before 2011 and this clemency should be considered by all parties involved (past and present).

The METC herewith formally states that the study on Urk is exempt of the notification obligation since it was carried out long before that time.
Upon request we wil confirm that this is an non-WMO study.

We hope we have informed you accordingly regarding this matter.

Kind regards,

Mrs drs. H.Tebbe
Secretary of the Isala METC
